# Revealing the range of equally likely estimates in the admixture model

**DOI:** 10.1093/g3journal/jkaf142

**Published:** 2025-06-19

**Authors:** Carola Sophia Heinzel, Franz Baumdicker, Peter Pfaffelhuber

**Affiliations:** Department of Mathematical Stochastics, Albert-Ludwigs-University Freiburg, Freiburg im Breisgau 79104, Germany; Cluster of Excellence “Controlling Microbes to Fight Infections”, Mathematical and Computational Population Genetics, University of Tübingen, Sand 14, Tübingen 72076, Germany; Institute for Bioinformatics and Medical Informatics (IBMI), University of Tübingen, Tübingen, 72074, Germany; Department of Mathematical Stochastics, Albert-Ludwigs-University Freiburg, Freiburg im Breisgau 79104, Germany

**Keywords:** admixture model, STRUCTURE, ADMIXTURE, nonunique estimator, label switching

## Abstract

Many ancestry inference tools, including Structure and Admixture, rely on the admixture model to infer both, allele frequencies *p* and individual admixture proportions *q* for a collection of individuals relative to a set of hypothetical ancestral populations. We show that under realistic conditions the likelihood in the admixture model is typically flat in some direction around a maximum-likelihood estimate $\left(\hat{q},\hat{p}\right)$. In particular, the maximum-likelihood estimator is nonunique and there is a complete spectrum of possible estimates. Common inference tools typically identify only a few points within this spectrum. We provide an algorithm which computes the set of equally likely $\left(\tilde{q},\tilde{p}\right)$, when starting from $\left(\hat{q},\hat{p}\right)$. It is analytic for K=2 ancestral populations and numeric for K>2. We apply our algorithm to data from the 1000 genomes project, and show that inter-European estimators of *q* can come with a large set of equally likely possibilities. In general, markers with large allele frequency differences between populations in combination with individuals with concentrated admixture proportions lead to small areas with a flat likelihood. Our findings imply that care must be taken when interpreting results from STRUCTURE and ADMIXTURE if populations are not separated well enough.

## Introduction

Inferring the ancestry of a sample of individuals from genetic data is a formidable challenge, given its importance in various domains, such as exploration of human history (Rosenberg *et al.* 2002), identification of missing persons (Phillips 2015), corrections for population stratification (Pritchard and Donnelly 2001), forensic genetics (Tvedebrink 2022), or conservation genetics (Wasser *et al.* 2007). In these applications, individual genomes are assumed to originate from different ancestral populations with distinct allele frequencies. Both, model-based methods and model-free techniques were developed for estimating individual admixture (Wollstein and Lao 2015). Model-based methods, such as Structure (Pritchard *et al.* 2000), Admixture (Alexander *et al.* 2009), and improvements of them (Falush *et al.* 2003, 2007; Hubisz *et al.* 2009; Alexander and Lange 2011; Raj *et al.* 2014; Shringarpure *et al.* 2016; Dominguez Mantes *et al.* 2023; Ko *et al.* 2023) treat the number of ancestral populations (usually denoted by *K*), allele frequencies within populations and individual admixture of all sample individuals as parameters of a statistical model, which we will call the Admixture model. Model-free methods use for example Principal Component Analysis or Spectral Graph Theory (Jombart *et al.* 2010; Lee *et al.* 2010; Klei *et al.* 2011) for inferring the individual ancestry (see Wollstein and Lao 2015 for an overview) and links between these two approaches have been made (Engelhardt and Stephens 2010). In recent years, neural networks have become yet another tool for inference of individual admixture, which follow the admixture model (Dominguez Mantes *et al.* 2023), or similar research questions (Battey *et al.* 2020).

There are various cases where the output of Structure and Admixture must be carefully interpreted. Different historic scenarios might lead to similar genetic data, and researchers have been warned not to over interpret estimates of the admixture model (Lawson *et al.* 2018). In addition, population structure is not correctly recovered in all cases (Puechmaille 2016), and it needs to be evaluated whether the assumptions of the admixture model are met, Garcia-Erill and Albrechtsen (2020). Another discussion in the admixture model comes from the choice of *K*. Since the *K* populations are ancestral, it is per se unclear what the right choice of *K* is, given an unstructured sample of genetic material (Pritchard *et al.* 2000; Evanno *et al.* 2005). As the admixture model does not account for the potential relationships between ancestral populations, it can result in contradictory predictions when using different values for *K* (Burger *et al.* 2024). Frequently, inferring *K* from the data lead to the choice K=2, see e.g. Janes *et al.* (2017), Friedlaender *et al.* (2008), Rosenberg *et al.* (2005), Lawson *et al.* (2018), Hamlin and Arnold (2014), and Phillips *et al.* (2009), while it is questioned if *K* can reasonably inferred at all (Verity and Nichols 2016; Wang 2017). Due to the increasing number of parameters, Structure is in practice not applied for many different populations, i.e. K≥10 (Lawson *et al.* 2012).

In the present study, we focus on principal limitations of the admixture model from a statistical perspective. Usually, Structure and Admixture result in maximum-likelihood estimators (or aposteriori estimators) of both, allele frequencies and admixture proportions. In a sample of *N* individuals, *M* bi-allelic markers and *K* ancestral populations, we have to estimate both, the N×K-matrix of individual admixtures, and the K×M-matrix of allele frequencies.

One of the well-known limitation of the admixture model is usually called *label-switching*, which means that relabeling ancestral populations leads to equivalent results. This corresponds to applying the same permutation to the population indices in both matrices. Label-switching is of practical importance, since it is one reason why different runs of Structure or Admixture can result in different estimates (Jakobsson and Rosenberg 2007; Alexander *et al.* 2009; Pritchard *et al.* 2010; Verdu *et al.* 2014; Kopelman *et al.* 2015; Behr *et al.* 2016; Cabreros and Storey 2019; van Waaij 2022; Liu *et al.* 2024). As a possible solution, a series of investigations tries to resolve label-switching with high computational effort in order to be able to compare different outputs of Structure (Rosenberg 2004; Jakobsson and Rosenberg 2007; Earl and VonHoldt 2012; Kopelman *et al.* 2015; Behr *et al.* 2016), even for different choices of *K* (Jia *et al.* 2020; Margaryan *et al.* 2020; Larena *et al.* 2021; Liu *et al.* 2024).

We show that the nonuniqueness of point estimates substantially extends beyond the phenomenon of mere label-switching and can be more severe. The differences between equally likely estimates are not just nuances, they can be substantial. However, the set of equally likely estimators is small if markers are able to distinguish clearly between populations. Therefore, our results highlight that reliable ancestry estimates depend on at least a few SNPs with large allele frequency differences between the populations. If such SNPs are unavailable, estimates within the admixture model must be taken with caution.

The structure of the paper is as follows: After introducing the admixture model, we start our investigation with a single estimator of individual admixtures and allele frequencies, and look for the range of estimators which are equally likely. From this, we can compute the differences between all the identified estimators with respect to the estimated allele frequencies and the estimated individual admixtures. This results in an algorithm, Emalam (Every MAximum-Likelihood estimator in the Admixture Model) available at our GitHub repository or as online version that explores the range of equally likely estimators. To show the consequences of the flatness of the likelihood curve, we apply our theoretical results to genetic data from the 1000 Genomes Project (The 1000 Genomes Project Consortium 2015). Furthermore, we compare our approach to Pong, a method proposed by Behr *et al.* (2016), i.e. to running Structure several times while accounting for label-switching to identify different modes. In contrast to the identification of the different modes discovered by STRUCTURE/ADMIXTURE, here we explore the complete space of equally likely estimators.

## Methods

### The admixture model

Let us reintroduce the admixture model in the unsupervised setting. For *M* markers, where marker *m* has $J_{m}$ different alleles, m=1,…,M, the genetic data of *N* diploid individuals are given by $x=(x_{ijm}{)}_{\left(i=1,\ldots ,N,j=1,\ldots ,J_{m},m=1,\ldots ,M\right)}$. Here, $x_{ijm}\in \{0,1,2\}$ determines the number of copies of allele *j* in individual *i* at locus *m*. We assume that we are dealing with diploid individuals, i.e. $\sum_{\;j=1}^{J_{m}}x_{ijm}=2$. We fix the number of distinct ancestral populations *K* and aim to infer the individual admixtures $q=(q_{ik}{)}_{i=1,\ldots ,N,k=1,\ldots ,K}$ and the allele frequencies $p=(p_{kjm}{)}_{k=1,\ldots ,K,j=1,\ldots ,J_{m},m=1,\ldots ,M}$ from the genetic data. Here, $q_{ik}$ represents the proportion of the genome of individual *i* that originates from population *k* (i.e. the individual ancestry of individual *i* in population *k*), and $p_{kjm}$ represents the frequencies of allele *j* in the assumed ancestral population *k* at marker *m*.

To depict the likelihood, we write $q_{i,\cdot }$ for the row vector $\left(q_{i1},\ldots ,q_{iK}\right)$, and the allele frequencies as $p_{\cdot jm}=(p_{1jm},\ldots ,p_{Kjm})$ and note that q,p satisfy the conditions (where $1_{K}$ is the 1-vector of length *K*)


(1) $$\begin{array}{cc} (\text{i})\;0\leq q_{ik}\leq 1, & \;(\text{ii})\;q_{i\cdot }1_{K}=1\\ (\text{iii})\;0\leq p_{kjm}\leq 1, & \;(\text{iv})\;p_{k\cdot m}1_{J_{m}}=1 \end{array}$$

i.e. the individual admixtures *q* of each individual sum to one and allele frequencies *p* sum to one at each locus. Given $x_{ijm}$, the number of alleles *j* of individual *i* at marker *m*, there is a constant $C_{x}$ which depends only on $x=(x_{ijm})$, such that the log-likelihood for p,q given *x* is, (using ⊤ to indicate column vectors)


(2) $$\ell (q,p|x)=C_{x}+\frac{1}{2MN}\sum_{i=1}^{N}\sum_{m=1}^{M}\sum_{\;j=1}^{J_{m}}x_{ijm}\log\;(q_{i\cdot }p_{\cdot jm}^{⊤}).$$

Here, $q_{i\cdot }p_{\cdot jm}^{⊤}$ is a scalar product, i.e. $q_{i\cdot }p_{\cdot jm}^{⊤}=\sum_{k=1}^{K}q_{ik}p_{kjm}.$ Apparently, the likelihood (2) depends on q,p only via $(q_{i\cdot }p_{\cdot jm}^{⊤}{)}_{i=1,\ldots ,N,j=1,\ldots ,J_{m},m=1,\ldots ,M}=qp$. Maximum-likelihood estimators are then given by


$$(\hat{q},\hat{p})\;:\;=({\hat{q}}_{x},{\hat{p}}_{x})\;:\;=\text{argmax}\{(q,p)\mapsto \ell (q,p|x)\}.$$

### Equally likely estimators

We start with an explanation why there are different estimators in the admixture model with the same likelihood. As stated above, the likelihood ℓ(q,p|x) depends on q,p only via the scalar products $q_{i\cdot }p_{\cdot jm}^{⊤}$. As a consequence, $\ell (\hat{q},\hat{p}|x)=\ell (\tilde{q},\tilde{p}|x)$ provided that


(3) $${\hat{q}}_{i\cdot }{\hat{p}}_{\cdot jm}^{⊤}={\tilde{q}}_{i\cdot }{\tilde{p}}_{\cdot jm}^{⊤}\;\text{ for all}\;i,j,m.$$

In order to see that this is in fact possible, let *S* be an invertible K×K matrix as well as


$${\tilde{q}}_{i\cdot }={\hat{q}}_{i\cdot }S,\;{\tilde{p}}_{\cdot jm}={\hat{p}}_{\cdot jm}(S^{-1}{)}^{⊤}.$$

Writing out the scalar product, ${\tilde{q}}_{i\cdot }{\tilde{p}}_{\cdot jm}^{⊤}={\hat{q}}_{i\cdot }S({\hat{p}}_{\cdot jm}S^{-1}{)}^{⊤}={\hat{q}}_{i\cdot }SS^{-1}{\hat{p}}_{\cdot jm}^{⊤}=$  ${\hat{q}}_{i\cdot }{\hat{p}}_{\cdot jm}^{⊤}$, i.e. (3) holds. However, we have to be careful in order not to violate the side conditions (1). In order to achieve this, restrict *S* to a matrix with rows summing to 1, i.e. $S1_{K}^{⊤}=1_{K}^{⊤}$. (If *S* has nonnegative entries, it is called a stochastic matrix.) Multiplying this equation by $S^{-1}$, it is clear that $S^{-1}1_{K}^{⊤}=1_{K}^{⊤}$ as well. Then, provided $\hat{q}$ and $\hat{p}$ satisfy (1), we have


$$\begin{array}{rl} {\tilde{q}}_{i\cdot }1_{K}^{⊤} & ={\hat{q}}_{i\cdot }S1_{K}^{⊤}={\hat{q}}_{i\cdot }1_{K}^{⊤}=1,\\ {\tilde{p}}_{k\cdot m}1_{J_{m}}^{⊤} & =\sum_{k^{\prime }}(S^{-1}{)}_{kk^{\prime }}{\hat{p}}_{k^{\prime }\cdot m}1_{J_{m}}^{⊤}=\sum_{k^{\prime }}(S^{-1}{)}_{kk^{\prime }}=1, \end{array}$$

i.e. $\tilde{q},\tilde{p}$ satisfy the side conditions (ii), (iv) as well. Moreover, if *S* is from


$$\begin{array}{rl} S_{\hat{q},\hat{p}} & \;:\;=\{S\;:\;S1_{K}=1_{K},{\hat{q}}_{i\cdot }S\geq 0\;\text{ for all}\;i,\\ & \;\sum_{k^{\prime }}{\hat{p}}_{k^{\prime }jm}(S^{-1}{)}_{kk^{\prime }}\geq 0\;\text{ for all}\;j,m\}, \end{array}$$

side conditions (i) and (iii) are satisfied for $\tilde{q},\tilde{p}$ as well. So, we have shown that the likelihood curve is flat in some directions of the parameter space, and hence likelihood-based estimators are not unique provided that $S_{\hat{q},\hat{p}}$ is not trivial. Moreover, we can search $S_{\hat{q},\hat{p}}$ for parameters which lead to equally likely individual admixtures and allele frequencies.

A well known but not exclusive example of this nonuniqueness deals with label-switching for populations. For this, consider a permutation matrix *S*. (Such a matrix has entries in {0,1} with a single 1 in each row/column.) Here, $Sq^{⊤}$ are individual admixtures where we have switched labels k→l if $(S{)}_{kl}=1$. Similarly, labels of allele frequencies *p*, switch label accordingly, i.e. using $S^{-1}p^{⊤}$. For such matrices, conditions (i), (iii) are always met and it has been discussed frequently (see e.g. Behr *et al.* 2016; Ko *et al.* 2023) that the estimators $Sq,S^{-1}p$ and q,p are equally likely. However, label switching is only one example, and the goal here is to identify the complete range of equally likely estimators beyond label switching.

For the interpretation of our results concerning the usage of STRUCTURE, there is another details we want to mention: The output of STRUCTURE is not a maximum-likelihood estimator. Rather, STRUCTURE samples the ancestries and the allele frequencies from the posterior distribution and outputs the mean of these samples. STRUCTURE uses an additional parameter, *α*, which can be used to calibrate the level of admixture in the prior distribution (Pritchard *et al.* 2000). However, in this study, we run STRUCTURE with the default parameters. Notice that choosing a uniform prior in combination with using the parameter INFERALPHA = 0 in STRUCTURE implies that the likelihood is proportional to the posterior distribution. Moreover, the influence of the prior usually decreases with the number of samples (Yuan and De Gooijer 2014), i.e. the prior distribution becomes negligible. An exception of this can e.g. be made by choosing the initial *α* according to Hubisz *et al.* (2009) suggestion, i.e. by using the sample location or the phenotype (option LOCPRIOR in STRUCTURE). Depending on the informativeness of the sampling location data, this can lead to a prior that helps to avoid a flat landscape of the posterior distribution in the admixture model.

### EMALAM

Our algorithm for exploring the set of equally likely estimators starts with a single estimator $\hat{q},\hat{p}$, as e.g. provided by the output of STRUCTURE or ADMIXTURE. We then search for $S\in S_{\hat{q},\hat{p}}$ as follows: Pick a subset I⊆{1,…,N} of individuals and (recalling that $H(x)=-\sum_{k}x_{k}\log\;x_{k}$ is the entropy of *x* which satisfies $x1^{⊤}=1$, which is maximal for the uniform distribution, and minimal for point measures) either


(4) $$\begin{array}{rl} & (\text{I})\;(a)\text{ minimize or }(b)\text{ maximize}\;\frac{1}{\left|I\right|}\sum_{i\in I}({\hat{q}}_{i\cdot }S{)}_{k}\;\text{ for some}\;k\\ & \text{or }(\mathrm{II})\;(a)\text{ minimize or }(b)\text{ maximize}\;\frac{1}{\left|I\right|}\sum_{i\in I}H({\hat{q}}_{i\cdot }S). \end{array}$$

So, in (I), we look at a concrete population *k* and minimize/maximize the average individual ancestry in *k* for all individuals in I. In (II), we rather minimize (favoring less admixed individuals) or maximize (favoring admixed individuals) the average entropy for individuals in I. See also Fig. 4 for an illustration how an equally likely estimator looks like for the four different options in an example data set.

### Nonidentifiability and allele frequency differences

**Fig. 1.**
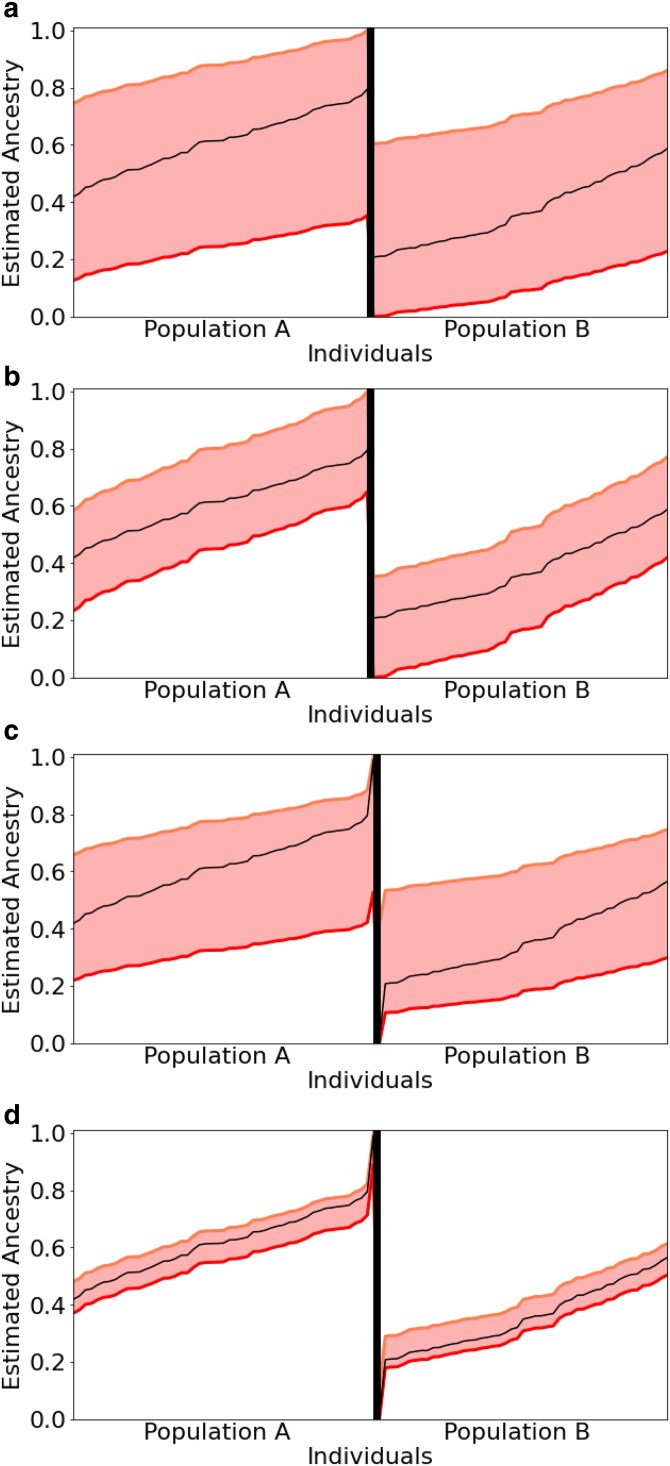
Range of equally likely estimators for two simulated populations. The *x*-axis represents the individuals and the *y*-axis indicates the individual ancestry for population A. The black lines show the true (simulated) individual ancestries. The area between the red lines symbolizes equally likely estimators.

Let us consider a toy example for the claimed nonidentifiability. We simulate 1000 markers with different allele frequencies among 100 admixed individuals from two populations. The true individual admixtures are depicted as the black line in Fig. 1, and the range of equally likely estimators as given by (5) are the colored area. Depending on whether we only have 1000 markers with intermediate allele frequency differences, or additionally two markers with a large frequency differential, the range of equally likely estimators varies; see Fig. 1. Recall that markers which are (almost) fixed in one, and polymorphic in all other populations, are called anchor markers. Equivalently, individuals with individual ancestry almost exclusively in one population, are called anchor individuals (Cabreros and Storey 2019). Figure 1 thus also illustrates how the addition of anchor markers and individuals influences the range of equally likely estimators. The estimated allele frequencies in population A for 1000 bi-allelic markers were simulated according to a uniform distribution in [0.25, 0.75]. The allele frequencies in population B are $p_{1,1,m}+\epsilon$, where ϵ is uniformly distributed in [0, 0.3] if $p_{2,1,m}\in [0.2,0.8]$ and $p_{2,1,m}=0.5$ else. Additionally, we added Fig. 1b and d two additional markers with frequencies (0.01,0.1),(0.9,0.99) in populations A and B, respectively. In Fig. 1c and d, we included two nonadmixed individuals with ancestry (0.01,0.99) and (0.99,0.01).

Without such anchors, the range of equally likely admixture proportions can span a substantial area such that the contribution of the two populations is basically impossible to disentangle (Fig. 1 top left). In the case with two populations A and B, if for an equally likely estimator the admixture proportion for population A of a certain individual is increased, so is the admixture proportion for population A in all other individuals, and the allele frequencies change accordingly. However, when K>2 the dependencies will be more complex.

### Data applications

Since our main focus is on a proof of concept of nonidentifiability in the admixture model, we use genetic data from the 1000 genomes project (The 1000 Genomes Project Consortium 2015). In our applications, we choose every 10,000th bi-allelic SNP among all SNPs with a minor allele frequency of 5%, resulting in about $10^{5}$ markers for intercontinental and about $7\cdot 10^{4}$ for intra-continental applications. Moreover, we considered the Kidd AIM set (Kidd *et al.* 2014) with 55 bi-allelic markers.

## Results

### Some theoretical insights

***The case***  K=2 is important from an application perspective (Rosenberg *et al.* 2005; Friedlaender *et al.* 2008; Phillips *et al.* 2009; Hamlin and Arnold 2014; Lawson *et al.* 2018). In this case, starting with an estimator $\hat{q},\hat{p}$, we give for each individual *i* the range of possible individual admixtures with the same likelihood.

We show in Theorem A1 in the Appendix that equally likely estimators for individual ancestry of individual *i* in population 1, i.e. $q_{i1}$, are in


(5) $$\left[{\hat{q}}_{i1}\frac{u^{*}-1}{u^{*}+v_{*}}+{\hat{q}}_{i2}\frac{1-u^{*}}{1+u^{*}/v_{*}};{\hat{q}}_{i1}\frac{1+v^{*}}{u_{*}+v^{*}}+{\hat{q}}_{i2}\frac{1+v^{*}}{1+v^{*}/u_{*}}\right]$$

with


$$\begin{array}{rl} {\hat{u}}_{*} & \;:\;=\min\left\{\frac{{\hat{p}}_{2jm}}{{\hat{p}}_{1jm}}\;:\;m=1,\ldots ,M,j=1,\ldots ,J_{m}\right\},\\ {\hat{u}}^{*} & \;:\;=\max\left\{\frac{{\hat{p}}_{2jm}}{{\hat{p}}_{1jm}}\;:\;m=1,\ldots ,M,j=1,\ldots ,J_{m}\right\},\\ {\hat{v}}_{*} & \;:\;=\min\left\{\frac{{\hat{q}}_{i1}}{{\hat{q}}_{i2}}\;:\;i=1,\ldots ,N\right\},\\ {\hat{v}}^{*} & \;:\;=\max\left\{\frac{{\hat{q}}_{i1}}{{\hat{q}}_{i2}}\;:\;i=1,\ldots ,N\right\}. \end{array}$$

Most notably, the range of possible admixture proportions of individual *i* among the equally likely estimators only depends on ${\hat{q}}_{i},{\hat{u}}_{*},{\hat{u}}^{*},{\hat{v}}_{*}$, and ${\hat{v}}^{*}$. So, adding markers $m^{\prime }$ or individuals $i^{\prime }$ with less extreme quotients ${\hat{p}}_{2jm^{\prime }}/{\hat{p}}_{1jm^{\prime }}$ and ${\hat{q}}_{i^{\prime }1}/{\hat{q}}_{i^{\prime }2}$ than markers/individuals already present in the dataset does not solve the problem of nonidentifiability per se. From the formula, the lower bound is near ${\hat{q}}_{i1}$ provided that both, $v_{*}\ll 1\ll u^{*}$, and the upper bound is close to ${\hat{q}}_{i1}$ iff $u_{*}\ll 1\ll v^{*}$. In other words, we need both, markers which can distinguish well between both populations, leading to $u_{*}\ll 1\ll u^{*}$, as well as individuals which are almost nonadmixed for both populations, leading to $v_{*}\ll 1\ll v^{*}$. This gives a more quantitative view on the fact that anchor individuals and anchor markers are necessary for uniqueness of the equally likely estimator; see the anchor conditions from Cabreros and Storey (2019). ***The case***  K>2 is more involved. Here, we did not obtain an analytical solution for the possible individual admixtures. However, we note that only changing $\hat{p}$ and $\hat{q}$ in two populations could be treated in the same way as the case K=2. However, this only leads to a lower bound for the set of possible individual admixtures. Therefore, we rely on numerical minimizations and maximizations in cases (I) and (II) for K>2 in order to find extreme, but equally likely estimators $\tilde{q},\tilde{p}$. Some more information can be found in the Documentation of EMALAM.

### Nonidentifiability in the 1000 genomes data

We apply Emalam to the data from the 1000 Genomes Project (The 1000 Genomes Project Consortium 2015). Additionally, we consider the difference between our method and running Structure many times as proposed by Behr *et al.* (2016).

In our workflow, we run Structure, and then study the range of equally likely estimators using the Structure estimator and EMALAM. We use data from northern Europeans from Utah (CEU, 99 individuals), Great Britain (GBR, 91 individuals), Iberian Populations in Spain (IBS, 107 individuals) and Tuscans from Italy (TSI, 107 individuals) in different combinations in the following. For the (small) marker set from Kidd *et al.* (2014), we make 20 independent runs of Structure, whereas we make only one run for the large marker set.

**Fig. 2.**
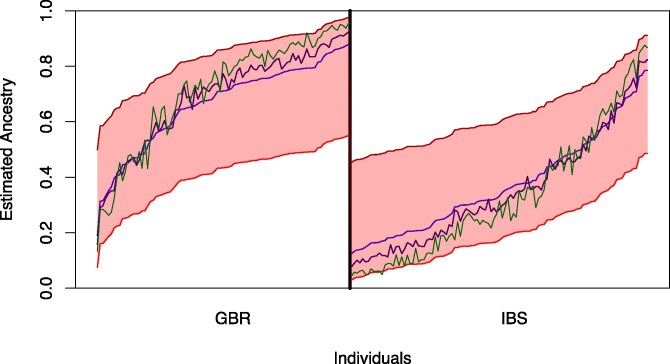
Range of equally likely estimators for GBR and IBS in the admixture model with K=2 with the marker set from Kidd *et al.* (2014). The colored area is between the minimal and maximal equally likely estimator for the individual ancestry, if we apply formula (5). The three colored lines depict the output of 20 runs of STRUCTURE. The software pong was used to only depict the three result modes that are not similar. Here, the green and dark blue line represent minor modes, while the blue line represents the major mode.

**Fig. 3.**
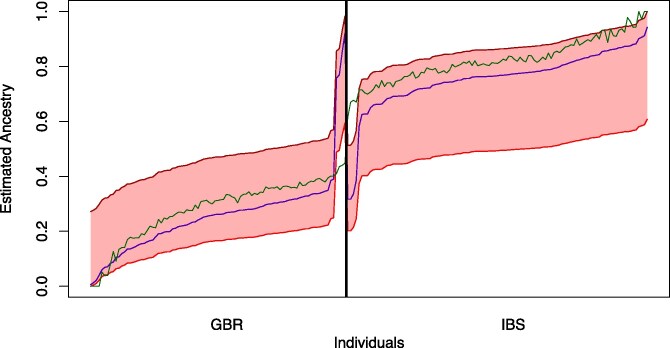
Range of equally likely estimators for GBR and IBS populations in the admixture model with K=2. Ancestry proportions of the estimators are depicted as in Fig. 2 but here we use 140,356 SNPs. We only consider bi-allelic SNPs, where the allele frequency of both alleles is higher than 0.05. Additionally, we used only every 10,000th SNP. The green line corresponds to the estimated ancestry of ADMIXTURE. The blue line is the output of STRUCTURE.

First, we consider two ancestral populations, K=2. Using only data from GBR and IBS, Figs. 2 and 3 show the range of equally likely individual admixtures (the colored area). In order to put our results into context, we (i) run Structure 20 times and apply pong (Behr *et al.* 2016) to the outputs to avoid label switching for the small marker set and (ii) compare the area of equally likely estimators when starting with the outputs of STRUCTURE and ADMIXTURE for the larger marker set. For (i), consider the blue and green lines in Fig. 2, which uses the marker set from Kidd *et al.* (2014). Here, the differences between the 20 runs of STRUCTURE are much smaller than the differences between the equally likely estimators found by EMALAM.

For (ii) consider the same individuals in Fig. 3, but using the large marker set of 71,185 SNPs. Here we see that the range of estimators with the same likelihood as the output of STRUCTURE is still large, comparable with the range when using the marker set from Kidd *et al.* (2014) (Fig. 2). We also see that the difference between the outputs of STRUCTURE and ADMIXTURE is small. Interestingly, we see that the difference between the maximal and the minimal estimator with the same likelihood as the output of ADMIXTURE is much smaller, i.e. so small that we cannot see any differences in Fig. 2.

**Fig. 4.**
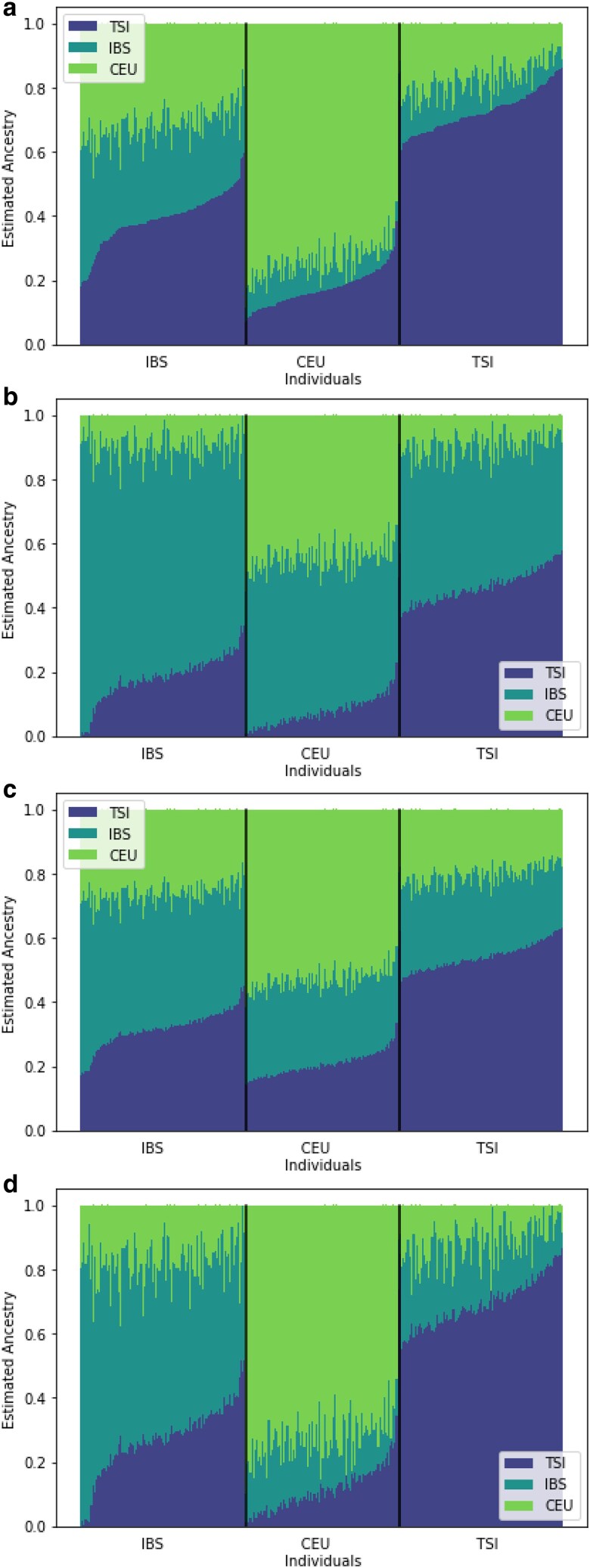
Estimated individual admixtures for the TSI, IBS, and CEU with 126,010 markers. We used EMALAM with K=3 in order to optimize the following target in the four figures: a) Minimize the ancestry from IBS (option (I)(a)), b) Maximize the ancestry from IBS (option (I)(b)), c) Minimize the entropy, i.e. option (II)(a), d) Maximize the entropy, i.e. option (II)(b).

Second, we consider another combination of populations, namely CEU, TSI and IBS where we apply Emalam to the data with K=3. In Fig. 4, the data were created by running Structure once and by applying option (I) (a) and (b) (recall from (4)) to the population IBS and option (II) (a) and (b) to the output of STRUCTURE for K=3. Especially, all four parts Fig. 4a–d show estimators with the same likelihood. Moreover, the order of individuals on the x-axis is the same for Fig. 4a–d. The different options of EMALAM aim to highlight different aspects where the interpretation of STRUCTURE and ADMIXTURE results can be particularly misleading when the complete range of estimators with equal likelihood is not considered. Option (I) maximizes and minimizes the ancestry proportions of a particular population, which can be useful to assess the confidence with which an individual is attributed to a certain bio-geographic background. Option (II) can highlight a different aspect as it maximizes or minimizes the entropy of the admixtures in all individuals. This option thus helps to assess the identifiability of separate populations and whether the individuals can be separated into multiple populations. This option helps determine if the estimators clearly suggest that individuals can be categorized into distinct populations or whether individuals are more admixed.

## Discussion

We demonstrated how the nonuniqueness of the estimators in the admixture model impacts the range of equally likely estimators.

Here, we introduced EMALAM, a method to explore the complete range of equally likely estimators. In addition, EMALAM is able to merge estimators that occur due to label switching; cf. pong (Behr *et al.* 2016). We stress that in general, the nonuniqueness of equally likely estimators has way more possibilities than just switching labels. Since EMALAM relies on a numerical minimization of a K×K-matrix, it is faster than running STRUCTURE or ADMIXTURE multiple times. Furthermore, it identifies the most extreme estimators with respect to specific aspects, i.e. the individual admixtures of a single individual or the admixture of a complete population.

In general, anchor markers, which have fixed alleles in only one population, and anchor individuals with nonadmixed ancestry, play a vital role in identifiability of the admixture model. If such anchors are missing, the range of estimators which EMALAM computes can be too large for a useful interpretation of the results provided by STRUCTURE or ADMIXTURE concerning the inference of the population structure, such that for some MLEs the ancestry fractions of one populations can vanish. Frequently, the lack of anchors goes hand in hand with choosing a too large number of ancestral populations. It is easy to see that the number of optima increases if *K* increases, i.e. in the admixture model *K* should be chosen as small as possible to prevent nonuniqueness. However, if suitable markers and individuals and a small enough *K* are considered the difference between equally likely estimators can be small (see Fig. 1).

EMALAM helps to investigate the range of equally likely estimators and how meaningful the inferred ancestries are. As a rule of thumb, small differences between the estimated individual ancestries and the estimated allele frequencies lead to a large range of possible estimated individual admixtures with the same likelihood as the output of Structure.

Our results highlight that a larger number of markers alone does not necessarily lead to a meaningful individual ancestry inference in the admixture model. It is crucial to include at least some markers with high allele differences between the populations of interest and to include for each population individuals with low admixture proportions. This implies that even when using thousands of markers (or individuals), the certainty of individual ancestry inference in the admixture model can rely on only a few ancestry informative markers (Fig. 1). Consequently, for populations where admixture occurs frequently or no informative markers exist the range of equally likely estimators in the admixture model can be larger than considering the different modes of multiple STRUCTURE runs suggests.

One caveat of applying EMALAM is that it only identifies equally likely estimators starting from a given initial estimator. Estimators with similar or even higher likelihoods are not detected, and the resulting range of equally likely estimators depends thus on the starting point. For example, if an estimator inferred by ADMIXTURE creates a spurious anchor individual or allele, the inferred range may appear narrow, which could potentially give a false impression of high confidence. While this is an extreme case, it is advisable to run STRUCTURE/ADMIXTURE multiple times to capture the variability in inferred modes, and apply EMALAM to each mode to better assess the range of equally likely estimators for each of them.

Using alternative models and methods that restrict the parameter space, e.g. by considering linkage disequilibrium and local ancestry (Falush *et al.* 2003; Corbett-Detig and Nielsen 2017), or by assuming dependency structures between the ancestral populations (Bradburd *et al.* 2018; Burger *et al.* 2024) can circumvent the nonidentifiability of the admixture model. However, while more complex models can lead to more precise individual admixtures and allele frequency estimates, they typically rely on a larger number of markers or specific model assumptions. Instead, EMALAM can be used to assess the consequences of the nonidentifiable equally likely estimators in applications based on the admixture model. Especially for scenarios with small allele frequency differences between populations EMALAM will help to prevent misinterpretation of ADMIXTURE and STRUCTURE results.

## Figures and Tables

**Fig. A1.**
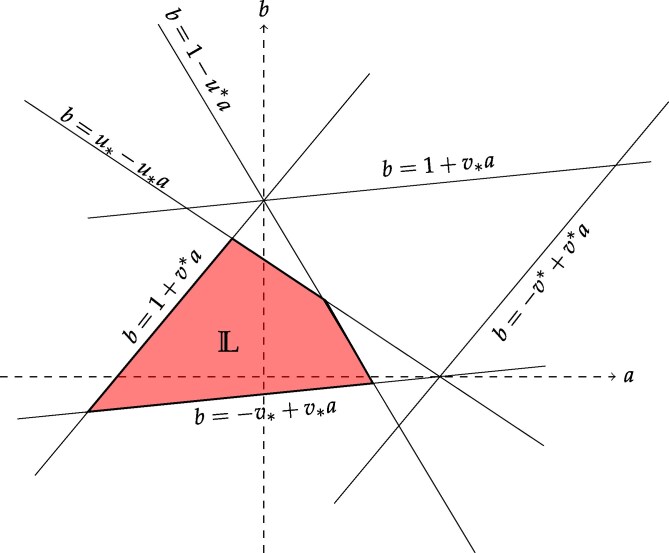
The set of (a,b), such that $\tilde{q}=\hat{q}S(a,b)$ and $\tilde{p}=\hat{p}(S(a,b{)}^{-1}{)}^{⊤}$ satisfies the conditions (1), is given by L.

**Fig. A2.**
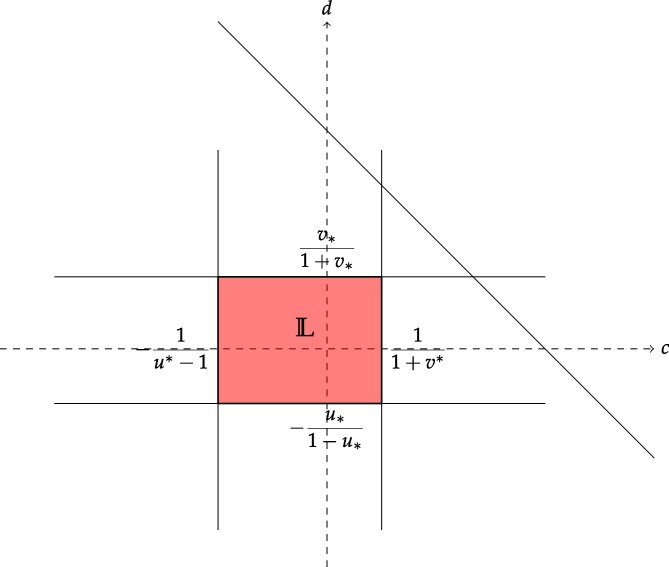
The set of (c,d), such that $\tilde{q}=\hat{q}S(a,b)$ and $\tilde{p}=\hat{p}(S(a,b{)}^{-1}{)}^{⊤}$ satisfies the conditions (1), is given by L.

## Data Availability

The data are available at the 1000 Genomes Project website (The 1000 Genomes Project Consortium 2015). The implementation of EMALAM can be downloaded from the GitHub repository (https://github.com/CarolaHeinzel/Flat-Likelihood-in-the-Admixture-Model). An online version is also available (https://flat-likelihood-in-the-admixture-model-p4vvpwxjdtteu3prahatyj.streamlit.app/).

## Appendix A: Theoretical results for two ancestral populations

Of course, K=2 in this section, so we suppress indices of 2 wherever possible. In addition, we assume we are given some $\hat{q}=({\hat{q}}_{i}{)}_{i=1,\ldots ,I}$, i.e. some estimator for all individual admixtures for all individuals, as well as $\hat{p}=({\hat{p}}_{kjm}{)}_{k=1,\ldots K,j=1,\ldots ,J_{m},m=1,\ldots ,M}$, i.e. all estimated allele frequencies at all markers satisfying (1). We will use vector notation whenever possible, e.g. p≥0 for some vector *p* means that all entries in *p* are ≥0. We start by noting that for $S\in S_{\hat{q},\hat{p}}$, we will require $\hat{q}S1^{⊤}=1^{⊤}$, which we achieve by assuming that $S1^{⊤}=1^{⊤}$, so we will work with *S* and the corresponding $S^{-1}$ of the form

$$\begin{array}{rl} S & =S(a,b)=\left(\begin{array}{cc} 1-a & a\\ b & 1-b \end{array}\right),\\ S^{-1} & =S^{-1}(a,b)=\frac{1}{1-a-b}\left(\begin{array}{cc} 1-b & -a\\ -b & 1-a \end{array}\right). \end{array}$$

For such *S*, we set

$$\tilde{q}\;:\;=\tilde{q}(a,b)=\hat{q}S(a,b),\;\tilde{p}=\hat{p}(S^{-1}(a,b){)}^{⊤}\;\text{or}\;{\tilde{p}}^{⊤}=S^{-1}(a,b){\hat{p}}^{⊤}.$$

Since $S(a,b)\in S_{\hat{q},\hat{p}}$ iff $S(1-a,1-b)=S(a,b)\cdot \left(\begin{array}{cc} 0 & 1\\ 1 & 0 \end{array}\right)\in S_{\hat{q},\hat{p}}$ (i.e. switching population labels leads to an additional solution), we prevent finding the solution with switched labels by using the condition a+b≤1, i.e. we do not consider the additional solution (1−a,1−b). So, our goal is to determine the set

$$L\;:\;=\{(a,b)\;:\;a+b\leq 1,\tilde{q}(a,b)\geq 0,\tilde{p}(a,b)\geq 0\}.$$

(Since summing *q* (over *k*) and *p* (over *j*) equals one as explained in Section 2, this suffices also for $\tilde{q}(a,b)\leq 1$ and $\tilde{p}\leq 1$.)

For the statement of our Theorem, we need the following notation:

$$\begin{array}{rl} {\hat{u}}_{*} & \;:\;=\min\left\{\frac{{\hat{p}}_{2jm}}{{\hat{p}}_{1jm}}\;:\;m=1,\ldots ,M,j=1,\ldots ,J_{m}\right\},\\ {\hat{u}}^{*} & \;:\;=\max\left\{\frac{{\hat{p}}_{2jm}}{{\hat{p}}_{1jm}}\;:\;m=1,\ldots ,M,j=1,\ldots ,J_{m}\right\},\\ {\hat{v}}_{*} & \;:\;=\min\left\{\frac{{\hat{q}}_{i1}}{{\hat{q}}_{i2}}\;:\;i=1,\ldots ,I\right\},\\ {\hat{v}}^{*} & \;:\;=\max\left\{\frac{{\hat{q}}_{i1}}{{\hat{q}}_{i2}}\;:\;i=1,\ldots ,I\right\}. \end{array}$$

Theorem A1
(Range for $\tilde{q}$ and $\tilde{p}$)
*The set L is as given in Fig. A1. Moreover, $\left(a^{*},b^{*}\right)$ and $\left(a_{*},b_{*}\right)$ maximizing and minimizing ${\tilde{q}}_{1}$ in all individuals are*

(A1 )
$$a^{*}=\frac{u_{*}-1}{u_{*}+v^{*}},\;b^{*}=\frac{1+v^{*}}{1+v^{*}/u_{*}},$$

(A2 )
$$a_{*}=\frac{1+v_{*}}{u^{*}+v_{*}},\;b_{*}=\frac{1-u^{*}}{1+u^{*}/v_{*}}.$$

*and for individual *i*, the resulting possible ${\tilde{q}}_{i1}$ are*

(A3 )
$${\tilde{q}}_{i1}\in \left[{\hat{q}}_{i1}\frac{u^{*}-1}{u^{*}+v_{*}}+{\hat{q}}_{i2}\frac{1-u^{*}}{1+u^{*}/v_{*}};{\hat{q}}_{i1}\frac{1+v^{*}}{u_{*}+v^{*}}+{\hat{q}}_{i2}\frac{1+v^{*}}{1+v^{*}/u_{*}}\right].$$

*In addition, the resulting possible ${\tilde{p}}_{kjm}$’s are*

(A4 )
$$\begin{array}{rl} & \text{between}\;{\hat{p}}_{1jm}\frac{u^{*}}{u^{*}-1}-\frac{1}{u^{*}-1}{\hat{p}}_{2jm}\\ & \text{and}\;-{\hat{p}}_{1jm}\frac{v^{*}}{1+v^{*}}+\frac{1}{1+v^{*}}{\hat{p}}_{2jm}\;\text{ for}\;{\tilde{p}}_{1jm},\\ & \text{between}\;-\frac{u_{*}}{1-u_{*}}{\hat{p}}_{1jm}+\frac{1}{1-u_{*}}{\hat{p}}_{2jm}\\ & \text{and}\;\frac{v_{*}}{1+v_{*}}{\hat{p}}_{1jm}+\frac{1}{1+v_{*}}{\hat{p}}_{2jm}\;\text{ for}\;{\tilde{p}}_{2jm}. \end{array}$$

Proof.

**Step 0: Computing**  L: As explained in Section 2, our task is to find *S* such that $\hat{q}S$ and $\hat{p}(S^{-1}{)}^{⊤}$ are nonnegative. So, for S=S(a,b), we find for $\hat{q}=({\hat{q}}_{1},{\hat{q}}_{2})$ and $\hat{p}=({\hat{p}}_{1},{\hat{p}}_{2})$ the conditions

(A5 ) $$\hat{q}S=({\hat{q}}_{1}(1-a)+b{\hat{q}}_{2},{\hat{q}}_{1}a+{\hat{q}}_{2}(1-b))\geq 0,$$

(A6 ) $$p(S^{-1}{)}^{⊤}=\frac{1}{1-a-b}({\hat{p}}_{1}(1-b)-a{\hat{p}}_{2},-b{\hat{p}}_{1}+{\hat{p}}_{2}(1-a))\geq 0.$$

From these equations, we find the equivalent conditions (note that we are using a+b≤1 in the second line)

$$\begin{array}{rl} & -\frac{{\hat{q}}_{1}}{{\hat{q}}_{2}}+\frac{{\hat{q}}_{1}}{{\hat{q}}_{2}}a\leq b\leq 1+\frac{{\hat{q}}_{1}}{{\hat{q}}_{2}}a\\ & b\leq \left(1-a\frac{{\hat{p}}_{2}}{{\hat{p}}_{1}}\right)\wedge \left(\frac{{\hat{p}}_{2}}{{\hat{p}}_{1}}-a\frac{{\hat{p}}_{2}}{{\hat{p}}_{1}}\right)=\left(1\wedge \frac{{\hat{p}}_{2}}{{\hat{p}}_{1}}\right)-a\frac{{\hat{p}}_{2}}{{\hat{p}}_{1}}. \end{array}$$

Since all conditions are linear, they are satisfied if and only if they hold for the maximal and minimal possible values for $\frac{{\hat{q}}_{1}}{{\hat{q}}_{2}}$ and $\frac{{\hat{p}}_{2}}{{\hat{p}}_{1}}$, i.e.

$$\begin{array}{rl} b & \geq -v_{*}+v_{*}a,\\ b & \geq -v^{*}+v^{*}a,\\ b & \leq 1+v_{*}a,\\ b & \leq 1+v^{*}a,\\ b & \leq u_{*}-au_{*},\\ b & \leq 1-au^{*}. \end{array}$$

We have used that ${\hat{u}}_{*}\leq 1$ and ${\hat{u}}^{*}\geq 1$ by construction: if ${\hat{p}}_{2jm}\leq {\hat{p}}_{1jm}$ for some j,m, there must be $j^{\prime }$ with ${\hat{p}}_{2j^{\prime }m}\geq {\hat{p}}_{1j^{\prime }m}$ since summing over *j* is 1. We have illustrated all inequalities and the resulting L in Fig. A1. Note that a+b≤1 is satisfied in L.
**Step 1: Maximizing and minimizing**  ${\tilde{q}}_{i1}$: We have to find combinations (a,b)∈L minimizing and maximizing ${\tilde{q}}_{i1}={\hat{q}}_{i1}(1-a)+b{\hat{q}}_{i2}$. In order to maximize ${\tilde{q}}_{i1}={\hat{q}}_{i1}(1-a)+b{\hat{q}}_{i2}$, note that a maximizer $(a^{*},b^{*})\in L$ must have the property that no $(a^{\prime },b^{\prime })\in L$ with $a^{\prime }<a^{*},b^{\prime }>b$ exists (since ${\tilde{q}}_{i1}(a^{\prime },b^{\prime })>{\tilde{q}}_{i1}(a^{*},b*)$ in this case, contradicting the maximization property of $\left(a^{*},b^{*}\right)$). This already implies that $\left(a^{*},b^{*}\right)$ is located on the line $b=1+v^{*}a$, since it is the line bounding L in the north-west. We find along this line

$$\begin{array}{rl} {\tilde{q}}_{i1}(a,1+v^{*}a) & ={\hat{q}}_{i1}(1-a)+(1+v^{*}a){\hat{q}}_{i2}\\ & ={\hat{q}}_{i1}+{\hat{q}}_{i2}+{\hat{q}}_{i2}\left(v^{*}-\frac{{\hat{q}}_{i1}}{{\hat{q}}_{i2}}\right)a. \end{array}$$

Since $v^{*}-\frac{{\hat{q}}_{i1}}{{\hat{q}}_{i2}}\geq 0$ by construction, $a\mapsto {\tilde{q}}_{i1}(a,1+v^{*}a)$ is increasing and the maximizer is at

$$u_{*}(1-a^{*})=1+v^{*}a^{*},$$

i.e.

$$\begin{array}{rl} a^{*} & =\frac{u_{*}-1}{u_{*}+v^{*}},\\ b^{*} & =1+v^{*}a^{*}=1+\frac{u_{*}v^{*}-v^{*}}{u_{*}+v^{*}}=\frac{u_{*}(1+v^{*})}{u_{*}+v^{*}}=\frac{1+v^{*}}{1+v^{*}/u_{*}}, \end{array}$$

which shows (A1). For the minimizer of ${\tilde{q}}_{i1}={\hat{q}}_{i1}(1-a)+b{\hat{q}}_{i2}$, we analogously note that a minimizer $(a_{*},b_{*})\in L$ must have the property that there is no $(a^{\prime },b^{\prime })\in L$ with $a^{\prime }>a^{*},b^{\prime }<b$. This implies that $\left(a_{*},b_{*}\right)$ is located on $b=-v_{*}+v_{*}a$, since this is the south-eastern boundary of L. We find

$${\tilde{q}}_{i1}(a,-v_{*}+v_{*}a)={\hat{q}}_{i1}(1-a)-v_{*}(1-a){\hat{q}}_{i2}={\hat{q}}_{i2}\left(\frac{{\hat{q}}_{i1}}{{\hat{q}}_{i2}}-v_{*}\right)(1-a).$$

Since $\frac{{\hat{q}}_{i1}}{{\hat{q}}_{i2}}\geq v_{*}$ by construction, $a\mapsto {\tilde{q}}_{i1}(a,1+v^{*}a)$ is decreasing and the minimizer $\left(a_{*},b_{*}\right)$ is at

$$-v_{*}(1-a_{*})=1-u^{*}a_{*},$$

i.e.

$$\begin{array}{rl} a_{*} & =\frac{1+v_{*}}{u^{*}+v_{*}},\\ & b_{*}=1-u^{*}a_{*}=1-\frac{u^{*}+u^{*}v_{*}}{u^{*}+v_{*}}=\frac{v_{*}(1-u^{*})}{u^{*}+v_{*}}=\frac{1-u^{*}}{1+u^{*}/v_{*}}, \end{array}$$

giving (A2). Plugging $\left(a^{*},b^{*}\right)$ into ${\tilde{q}}_{i1}={\hat{q}}_{i1}(1-a^{*})+b^{*}q_{i2}$, we obtain the maximal ${\tilde{q}}_{i1}$, which is given by

$${\tilde{q}}_{i1}={\hat{q}}_{i1}\frac{1+v^{*}}{u_{*}+v^{*}}+{\hat{q}}_{i2}\frac{1+v^{*}}{1+v^{*}/u_{*}}.$$

Accordingly, we obtain the minimal ${\tilde{q}}_{i1}$ as

$${\tilde{q}}_{i1}={\hat{q}}_{i1}\frac{u^{*}-1}{u^{*}+v_{*}}+{\hat{q}}_{i2}\frac{1-u^{*}}{1+u^{*}/v_{*}}$$

This gives (A3) and we have shown all claims for the range of $\tilde{q}$. **Step 2: Maximizing and minimizing**  ${\tilde{p}}_{kjm}$: Here, we have to find combinations (a,b)∈L minimizing and maximizing ${\tilde{p}}_{1jm}=\frac{1-b}{1-a-b}{\hat{p}}_{1jm}-\frac{a}{1-a-b}{\hat{p}}_{2jm}$ and ${\tilde{p}}_{2jm}=\frac{-b}{1-a-b}{\hat{p}}_{1jm}+\frac{1-a}{1-a-b}{\hat{p}}_{2jm}$. We start by a reparameterization of *S* with

$$S^{-1}=\left(\begin{array}{cc} 1-c & c\\ d & 1-d \end{array}\right),\;S=\frac{1}{1-c-d}\left(\begin{array}{cc} 1-d & -c\\ -d & 1-c \end{array}\right).$$

Since we restrict to a+b≤1, we must assume

$$\frac{-c-d}{1-c-d}\leq 1\;\text{or}\;1-\frac{-c-d}{1-c-d}=\frac{1}{1-c-d}\geq 0,$$

which implies c+d≤1. So, we impose the restriction c+d≤1 in the sequel. In other words, we set

$$c=\frac{a}{1-a-b},\;d=\frac{b}{1-a-b},$$

or

$$a=\frac{c}{1+c+d},\;b=\frac{d}{1+c+d}.$$

The conditions (A5) and (A6) become

$$\begin{array}{rl} \hat{q}S & =\frac{\left({\hat{q}}_{1}-d,{\hat{q}}_{2}-c\right)}{1-c-d}\geq 0,\\ \hat{p}(S^{-1}{)}^{⊤} & =({\hat{p}}_{1}(1-c)+c{\hat{p}}_{2},d{\hat{p}}_{1}+(1-d){\hat{p}}_{2})\geq 0. \end{array}$$

Since c+d≤1, we have

$$\begin{array}{rl} d & \leq {\hat{q}}_{1},c\leq {\hat{q}}_{2},\\ c & \leq \frac{{\hat{p}}_{1}}{{\hat{p}}_{1}-{\hat{p}}_{2}}=\frac{1}{1-{\hat{p}}_{2}/{\hat{p}}_{1}}\;\text{if}\;{\hat{p}}_{1}>{\hat{p}}_{2},\\ c & \geq -\frac{1}{{\hat{p}}_{2}/{\hat{p}}_{1}-1}\;\text{if}\;{\hat{p}}_{1}<{\hat{p}}_{2},\\ d & \leq \frac{{\hat{p}}_{2}}{{\hat{p}}_{2}-{\hat{p}}_{1}}=\frac{1}{1-{\hat{p}}_{1}/{\hat{p}}_{2}}\;\text{if}\;{\hat{p}}_{2}>{\hat{p}}_{1},\\ d & \geq -\frac{1}{{\hat{p}}_{1}/{\hat{p}}_{2}-1}\;\text{if}\;{\hat{p}}_{2}<{\hat{p}}_{1}. \end{array}$$

The first three inequalities have to hold for all *i*, so we can write

$$\begin{array}{rl} d & \leq \min{\hat{q}}_{i1}=\frac{v_{*}}{1+v_{*}},\\ c & \leq \min{\hat{q}}_{i2}=\frac{1}{1+v^{*}}. \end{array}$$

The latter four inequalities have to hold for all j,m, we can replace them by

$$\begin{array}{rl} c & \leq \frac{1}{1-u_{*}},\\ c & \geq -\frac{1}{u^{*}-1},\\ d & \leq \frac{1}{1-1/u^{*}},\\ d & \geq -\frac{1}{1/u_{*}-1}. \end{array}$$

An illustration of all points (c,d) satisfying these inequalities is given in Fig. A2. In order to maximize (or minimize) ${\tilde{p}}_{1jm}$, we have to distinguish two cases. If ${\hat{p}}_{2jm}>{\hat{p}}_{1jm}$, we have to find the maximal (minimal) allowed *c*. If ${\hat{p}}_{2jm}<{\hat{p}}_{1jm}$, we must rather find the minimal (maximal) allowed *c*. In each case, ${\tilde{p}}_{1jm}$ is between

$${\hat{p}}_{1jm}\frac{u^{*}}{u^{*}-1}-\frac{1}{u^{*}-1}{\hat{p}}_{2jm}\;\text{and}\;-{\hat{p}}_{1jm}\frac{v^{*}}{1+v^{*}}+\frac{1}{1+v^{*}}{\hat{p}}_{2jm}.$$

Similarly, ${\tilde{p}}_{2jm}$ is between

$$-\frac{u_{*}}{1-u_{*}}{\hat{p}}_{1jm}+\frac{1}{1-u_{*}}{\hat{p}}_{2jm}\;\text{and}\;\frac{v_{*}}{1+v_{*}}{\hat{p}}_{1jm}+\frac{1}{1+v_{*}}{\hat{p}}_{2jm}.$$

This gives (A4) and we have shown all claims.
